# Supplementary material for: Specialty palliative care use among cancer patients: A population-based study
Source: PLoS One. 2025 Jan 13;20(1):e0313732. doi: 10.1371/journal.pone.0313732 (PMC11730419; doi:10.1371/journal.pone.0313732)
Supplement: S1 Table — (DOCX) [file pone.0313732.s001.docx]

**S1 Table. Logistic regression of SPC use among those (n=6,707) who went to a hospital offering SPC in final six months of life.** AUC=0.694. SPC = specialty palliative care; SES = socioeconomic status; OR = odds ratio; CI = confidence interval.

| Characteristics | OR (95% CI) |
| --- | --- |
| SES |  |
| Rank 2 v 1 | 1.24 (1.03, 1.48) |
| Rank 3 v 1 | 1.11 (0.93, 1.33) |
| Rank 4 v 1 | 1.23 (1.03, 1.48) |
| Rank 5 v 1 | 1.36 (1.13, 1.64) |
| Age at death (10-year increments) | 0.89 (0.85, 0.93) |
| Black | 1.38 (1.21, 1.56) |
| Any hematological malignancy | 0.73 (0.62,0.85) |
| Years from diagnosis to death | 0.95 (0.91, 0.99) |
| Death year |  |
| 2013 vs 2012 | 1.09 (0.93, 1.28) |
| 2014 vs 2012 | 1.25 (1.07, 1.46) |
| 2015 vs 2012 | 1.38 (1.18, 1.61) |
| Rurality group |  |
| 1-50% vs 0% rural | 1.04 (0.89, 1.21) |
| 51-99% vs 0% rural | 0.95 (0.73, 1.22) |
| 100% vs 0 % rural | 1.33 (1.13, 1.55) |
| Any admits 30 days prior to PC or death | 11.30 (9.06,14.10) |
| Low SES insurance | 1.45 (1.20, 1.76) |
