# Supplementary material for: Specialty palliative care use among cancer patients: A population-based study
Source: PLoS One. 2025 Jan 13;20(1):e0313732. doi: 10.1371/journal.pone.0313732 (PMC11730419; doi:10.1371/journal.pone.0313732)
Supplement: S2 Table — (DOCX) [file pone.0313732.s002.docx]

**S2 Table. Logistic regression of SPC use among those with poor prognosis cancer or stage (n=4,780).** AUC=0.723. SPC = specialty palliative care; SES = socioeconomic status; OR = odds ratio; CI = confidence interval.

| Characteristics | OR (95% CI) |
| --- | --- |
| Age at death (10-year increments) | 0.81 (0.76, 0.86) |
| Black | 1.36 (1.17, 1.59) |
| Years from diagnosis to death | 0.83 (0.73, 0.94) |
| Death year |  |
| 2013 vs 2012 | 0.99 (0.80, 1.22) |
| 2014 vs 2012 | 1.18 (0.96, 1.45) |
| 2015 vs 2012 | 1.57 (1.28, 1.93) |
| Rurality group |  |
| 1-50% vs 0% rural | 1.12 (0.92, 1.38) |
| 51-99% vs 0% rural | 0.58 (0.41, 0.84) |
| 100% vs 0 % rural | 0.91 (0.74,1.11) |
| Any admits 30 days prior to PC or death | 9.02 (7.00,11.62) |
